# Gadolinium- and water-based blood-brain barrier dysfunction measures in patients with sporadic small vessel disease

**DOI:** 10.1016/j.cccb.2026.100528

**Published:** 2026-01-13

**Authors:** Michael S Stringer, Xingfeng Shao, Hedok Lee, Antoine Vallatos, Carmen Arteaga-Reyes, Una Clancy, Francesca Chappell, Cameron Manning, Maria Valdes-Hernandez, Daniela Jaime Garcia, Rosalind Brown, Fergus N Doubal, Helene Benveniste, Michael J Thrippleton, Danny JJ Wang, Joanna M Wardlaw, Mild Stroke Study 3 study group

**Affiliations:** a Centre for Clinical Brain Sciences and UK Dementia Research Institute, University of Edinburgh, Edinburgh, United Kingdom; b School of Cardiovascular & Metabolic Health, University of Glasgow, Glasgow, United Kingdom; c Laboratory of FMRI Technology (LOFT), USC Mark & Mary Stevens Neuroimaging and Informatics Institute, Keck School of Medicine, University of Southern California, Los Angeles, CA, USA; d Department of Anesthesiology, Yale School of Medicine, New Haven, CT, USA; e Glasgow Experimental MRI Centre, School of Psychology and Neuroscience, University of Glasgow, Glasgow, United Kingdom; f Department of Biomedical Engineering, Yale School of Medicine, New Haven, CT, USA

**Keywords:** Blood-brain barrier, Water exchange rate (*k_w_*), small vessel disease, Stroke, MRI, Cerebrovascular disease

## Abstract

**Background:**

Subtle blood-brain barrier (BBB) leakage has been detected in small vessel disease (SVD). While established methods rely on gadolinium-based contrast agents (GBCA), diffusion-weighted arterial spin labelling (DW-ASL) is a promising alternative which assesses water exchange rate (*k_w_*) without injected contrast. However, DW-ASL has not been widely applied in sporadic SVD. We aimed to determine how *k_w_* varied with GBCA BBB leakage measures, baseline and 1-year change in SVD burden.

**Methods:**

We recruited patients with mild ischaemic stroke (lacunar or cortical) all characterised for SVD features. We assessed *k_w_* using DW-ASL and GBCA measures of BBB leakage (permeability-surface area product (*PS*), blood plasma volume and exchange rate of GBCA) using dynamic-contrast enhanced MRI. We used separate linear regression models to assess how *k_w_* varied with GBCA-derived metrics, baseline and 1-year change in WMH volume in co-variate-adjusted analyses.

**Results:**

We included 24 with complete MRI (61±10 years; 71% male). Patients with higher *k_w_* tended to have more severe baseline SVD (e.g. subcortical grey matter (SGM): B=14.59 min^-1^/%WMH volume, 95% confidence interval (95%CI)=-1.00,28.18, p=0.04) and greater 1-year increase (B=0.0013 %WMH volume increase/min^-1^, 95%CI=-0.0001, 0.0026, p=0.06). We generally found *k_w_* and GBCA BBB leakage measures were not meaningfully associated (e.g. SGM *k_w_*∼*PS*: B=-0.23 min^-1^/10^-4^ min^-1^, 95%CI=-7.47, 7.01, p=0.95).

**Conclusion:**

BBB water exchange estimated using DW-ASL tended to be greater with higher WMH burden and progression, suggesting *k_w_* may be a sensitive measure of BBB dysfunction in SVD. However, non-concordance between *k_w_* and GBCA metrics suggests the two methods probe different aspects of BBB function.

## Highlights

• Worse sporadic small vessel disease (SVD) burden linked to higher water exchange.
• 1-year small vessel disease increase also associated with higher water exchange.
• Water exchange rate was sensitive to subtle blood-brain barrier changes in SVD.

## Introduction

Cerebral small vessel diseases (SVDs) cause one quarter of ischaemic strokes and up to a half of dementias, either vascular or mixed, typically begins covertly and increases with age, often being identified incidentally [1]. However, symptom presentation can be broad, including cognitive impairment, problems with mobility or altered mood [2].

The pathophysiology of SVD remains poorly understood, though blood-brain barrier (BBB) dysfunction is thought to play an important role [1]. Brain damage may be caused by several BBB dysfunction components, including impaired endothelial cell dysfunction, fluid, protein and fibrinogen leakage into the walls of blood vessels leading to aberrant thickening and stiffening of arteriole walls, impairing vasoreactivity, oxygen and nutrient transport, perivascular oedema and neuroinflammation [1,3]. While subtly increased with normal ageing [4], BBB leakage is higher in patients with Alzheimer’s disease, worse in patients with SVD who have higher white matter hyperintensity (WMH) burden [3,5]. and steeper post-stroke cognitive decline [6].

In vivo BBB leakage can be evaluated using MRI [3]. Dynamic-contrast enhanced MRI (DCE-MRI), which tracks T1-weighted signal enhancement over time following intravenous injection of a Gd-based contrast agent (GBCA), is the most widely used approach. Using pharmacokinetic models, BBB leakage can be quantitatively assessed [7]. In populations with subtle BBB leakage, the Patlak model, which assesses the permeability-surface area product (*PS*) and blood plasma volume fraction (*v_P_*), is favoured [3,8]. However, intravenous injection is required, compromised kidney function is a contraindication, and concerns about possible damage due to Gd retention limits has increased resistance to repeated scanning and the large contrast agent molecular size limits sensitivity [3,9]. Therefore, several techniques using water as an endogenous contrast agent have been developed, including diffusion-weighted arterial spin labelling (DW-ASL) which proposes to measure the water exchange rate (*k_w_*) across the microvessel wall [10]. However, findings in studies relevant to SVD have varied. In previous work, higher whole brain *k_w_* correlated with higher Fazekas scores in community dwelling older subjects [9], but, relative to age- and sex-matched controls, *k_w_* was lower in genetic forms of SVD which have a severe SVD lesions (e.g. Cerebral Autosomal Dominant Arteriopathy with Subcortical Infarcts and Leukoencephalopathy (CADASIL) and high‐temperature requirement factor A serine peptidase 1 (HTRA1)‐related SVD) [11]. Lower *k_w_* was associated with worse cognitive function in genetic SVD and across the Alzheimer’s disease continuum [12], and lower CSF Aβ42 concentrations [13]. However, DW-ASL has not been widely applied in patients with stroke-related SVD e.g. lacunar stroke or in non-genetic forms of WMHs [14] and no studies have assessed longitudinal associations prospectively [15,16]. Moreover, to our knowledge, only 2 studies have contemporaneously assessed *k_w_* and GBCA BBB leakage, finding limited region-dependent associations, likely as water and GBCA transport mechanisms differ, but only one assessed symptomatic sporadic SVD patients [14].

Here we compared DW-ASL and DCE-MRI measures of BBB function in the same participants in a prospective, longitudinal study of well-characterised patients with symptomatic SVD. We assessed associations between water exchange and age, SVD burden and 1-year change. We hypothesised that *k_w_* and *PS* would have limited inter-associations but would independently associate with WMH volume and 1-year change.

## Methods

### Participants and study procedure

As previously described in our published protocol [17], we prospectively recruited patients with mild ischaemic stroke (defined as modified NIH Stroke Secale [NIHSS] <8 and expected to be non-disabling i.e. modified Rankin Score [mRS]≤2) presenting at Edinburgh/Lothian stroke services, United Kingdom (Mild Stroke Study 3, ISRCTN:12113543). Stroke diagnosis and subtype was determined by specialist stroke physicians and neuroradiologists. In the parent study, we included patients with lacunar ischaemic stroke (clinically symptomatic SVD) and cortical ischaemic stroke, as controls for having a stroke, common risk factors, and usual medications. The inclusion of cortical ischaemic stroke also allowed us to include a broader range of SVD features. Detailed inclusion and exclusion criteria and study procedures have been previously published [17]. In brief, we excluded patients with contraindications to MRI, other major neurological conditions, and severe cardiac and respiratory diseases. Data can be made available upon reasonable request to the senior author (J.M.W.).

We invited patients within 1-3 months of stroke onset to avoid acute effects of the infarct on BBB leakage. We assessed stroke severity (NIHSS) and a medical history, including vascular risk factors, was taken by a medical doctor (U.C./C.A.R) overseen by one expert consultant stroke physician (F.D.). We measured blood pressure and acquired brain MRI scans for each patient.

### Brain MRI acquisition

At baseline, we scanned all participants on a 3 T MRI scanner (MAGNETOM Prisma, Siemens Healthcare, Erlangen, Germany). The full image acquisition protocol has been previously described [17]. In brief, we acquired:

• Structural images to assess SVD burden (3D T1-weighted, T2-weighted, FLAIR, susceptibility weighted imaging (SWI) and diffusion MRI)
• Quantitative T1 (qT1) mapping (two inversion recovery (IR-) spoiled gradient recalled echo (SPGR): TR/TE/TI=1040/1.82/600 and 1940/1.82/1500 ms, FA=5°; three SPGR: TR/TE = 5.4/1.82 ms, FA = 2/5/12°, acquisition matrix 160 × 200 × 160, 1.2-mm isotropic)
• DCE-MRI (32 consecutive SPGR volumes during intravenous injection of 0.1 mmol/kg body weight gadobutrol (1M Gadovist, Bayer AG, Leverkusen, Germany) using a power injector; TR/TE = 3.4/1.7 ms, FA = 15°, acquisition matrix size 120 × 96 × 96, 2-mm isotropic)

Additionally, for this exploratory pilot study, in a subset of consecutive patients and prior to gadolinium-based contrast agent (GBCA) injection, we acquired a diffusion-prepared pseudocontinuous arterial spin labelling (DP-pCASL) sequence (TR/TE=4000/36.5 ms, FA=120°, acquisition matrix=64 × 64, 12 axial slices (10% oversampling), 3.5 × 3.5 × 8 mm^3^ resolution, label/control durations=1500 ms, post-labelling delay (PLD)=900 and 1800 ms and b=0 and 50 s/mm^2^ for the PLD=1800 ms scan) [9].

After one-year, all patients returned for a follow-up visit which included repeat structural imaging using the same 3T MRI scanner and imaging protocol (3D T1-w, T2-w, FLAIR, SWI, diffusion MRI and qT1) as at baseline.

### Structural MRI analysis

We performed all analyses of structural scans blinded to clinical information and BBB dysfunction measures. Trained raters visually assessed all structural MRI scans for index and incident infarcts and key SVD features under supervision of an experienced neuroradiologist using the STRIVE-1 criteria including deep and periventricular WMH severity using the Fazekas score, basal ganglia and centrum semiovale perivascular space score, number of lacunes and microbleeds [18].

For computational analyses, we registered all structural images to the baseline T2-w scan using FSL-FLIRT (FMRIB Software Library, FMRIB Analysis Group, Oxford, United Kingdom) [19]. Using previously described computational methods, highly validated for use in stroke and other SVD-related populations, we segmented WMH on the registered FLAIR image [20]. We generated a brain mask based on the coregistered T1-w, T2-w and FLAIR images. We segmented subcortical grey (SGM) and whole-brain normal-appearing white matter (NAWM) using an in-house pipeline combining FreeSurfer and FSL-FAST outputs [20]. One researcher, supervised by an expert neuroradiologist, manually segmented acute index stroke lesions and subsequent incident infarcts based on the registered FLAIR image. We excluded the manually segmented stroke lesions from the tissue masks, checked and manually corrected all masks as necessary [17]. We calculated percentage white matter volume normalised to intracranial volume (ICV).

### DCE-MRI processing

We processed the quantitative T1 and DCE-MRI data in MATLAB (MathWorks, MA, USA) using in-house code as previously described [21] and following consensus recommendations [3]. We measured pre-contrast T1 maps using the DESPOT1-HIFI method [22], correcting flip angle error voxelwise [23]. For the DCE-MRI, we manually determined a patient-specific venous input function by selecting five voxels in the superior sagittal sinus and calculating the mean signal time-course. We calculated signal enhancement timecourses relative to the mean pre-contrast signal using the median signal within each tissue mask [21]. We calculated SGM, NAWM and WMH permeability surface area product (*PS*) and blood plasma volume (*v_P_*) from the median tissue enhancement timecourses using an unconstrained multiple linear regression implementation of the Patlak model [21,24]. For comparability and to attempt to partially correct for differences in vascularity, we computed the exchange rate of GBCA (*k_Gad_*) as the ratio between *PS* and *v_P_* [9].

### DW-ASL processing

We used a two-stage approach to measure arterial transit time (ATT) and water exchange rate (*k_w_*) [25]. We calculated *k_w_* using a total-generalized variation regularized single-pass-approximation model from scans acquired at PLD=1,800 ms with b=0 and 50 s/mm^2^, intended to separate the intra- and extra-vascular compartments, respectively [9].

### Statistics

We performed all statistical analyses in R (v4.2.3, https://cran.r-project.org) and reported all data for all available participants. We calculated summary statistics as the mean ± standard deviation.

We assessed differences between tissue types in *k_w_, PS, v_P_* and *k_Gad_* using 1-way paired ANOVAs. Where we found meaningful differences, we used paired 2-sample t-tests to compare how SGM and WMH differed relative to NAWM.

We investigated associations between *k_w_* and *PS, v_P_* and *k_Gad_* (*PS*/*v_P_*) in separate models using univariate and multiple variable linear regression adjusted for age and percentage WMH volume (as a marker of SVD burden).

We evaluated whether SGM, NAWM and WMH *k_w_, PS, v_P_* and *k_Gad_* differed with percentage WMH volume using separate multiple linear regression for each tissue type and BBB variable adjusted for age.

Finally, we assessed associations between 1-year change in percentage WMH volume (outcome) and *k_w_, PS, v_P_* and *k_Gad_* in separate multiple linear regression models adjusted for baseline WMH volume and age.

For all analyses, we checked underlying statistical assumptions, including checking for collinearity using variance inflation factors. We scaled *PS* (x10,000) and *v_P_* (x100) for range consistency with other variables. We reported all linear regressions as unstandardised coefficients (B), 95% confidence intervals and p-value. Consistent with the American Statistical Society’s Statement on p-value [26,27], we interpreted relationships between variables based on the point estimate directions of effect, confidence interval breadth and existing clinical knowledge rather than only p-values. As this is an exploratory analysis, we did not apply any correction for multiple comparisons.

## Results

We recruited 26 patients. All patients had complete processable baseline structural and DCE-MRI scans, but two DW-ASL scans failed. At 1-year follow-up, 22 of the 24 patients had structural MRI data (1: claustrophobia, 1 unable to attend).

The remaining 24 patients had a mean age of 61±10 years, and 71% were male, 71% had hypertension, 67% hypercholesterolaemia, 17 % diabetes, 12% atrial fibrillation and 50% were current/ex-smokers (Table 1). Participants had a mean percentage WMH/ICV volume of 0.72±0.66%.

**Table 1:** Patient demographic and clinical variables. Categorical variables are given as number (percentage) with diagnostic variables coded as 1 and 0 for diagnosed/not diagnosed. Continuous numeric variables are quoted as mean ± standard deviation (SD), Fazekas score as median ± interquartile range (WMH=white matter hyperintensity).

	Total (N=24)
**Sex**	
Male	17 (71 %)
Female	7 (29 %)
**Age (years)**	
Mean ± SD	61 ± 10
**Stroke Subtype**	
Lacunar	13
Cortical	11
**Systolic BP (mmHg)**	
Mean ± SD	148 ± 23
**NIHSS score at baseline**	
Mean ± SD	0.79 ± 0.66
**Hypertension**	
No	7 (29 %)
Yes	17 (71 %)
**Diabetes**	
No	20 (83 %)
Yes	4 (17 %)
**Atrial Fibrillation**	
No	21 (88 %)
Yes	3 (12 %)
**Hypercholesterolaemia**	
No	8 (33 %)
Yes	16 (67 %)
**Smoking History**	
Never	12 (50 %)
Current/Ex	12 (50 %)
**Fazekas score**	4 (3-6)
**Percentage WMH volume (%)**	
Baseline Mean ± SD	0.743 ± 0.661
1-year^a^ Mean ± SD	0.749 ± 0.680

a) n=22, 2 participants did not have 1-year follow-up scans (n=1, claustrophobia; n=1, unable to attend).

We did not find measurable differences between tissues (SGM, NAWM or WMH) in *k_w_* (F=0.06, p=0.94) or *k_Gad_* (F=0.28, p=0.76). However, *PS* and *v_P_* in NAWM tended lower relative to SGM (*PS*: t=-3.24, p=0.002; *v_P_*: t=-8.91, p<0.001) and WMH (*PS*: t=-1.86, p=0.07; *v_P_*: t=-2.36, p=0.02). Table 2 and Figure S1 show mean values and distribution of the imaging variables.

**Table 2:** Blood-brain barrier variables calculated from dynamic-contrast enhanced MRI and diffusion-weighted arterial spin labelling in normal-appearing white matter (NAWM), subcortical grey matter (SGM) and white matter hyperintensities (WMH).

	Total			
	NAWM	SGM	WMH^a^	One-way
	(N=24)	(N=24)	(N=24)	paired ANOVA
**Water exchange rate (min^-1^)**				
Mean ± SD	115.56 ± 23.79	117.87 ± 21.01	115.80 ± 30.10	F=0.06,
				p=0.94
**Permeability surface area product (10^-4^min^-1^)**				
Mean ± SD	0.44 ± 1.17	1.57 ± 1.23	1.16 ± 1.47	F=4.61,
				p=0.01
**Blood plasma volume (10^-2^)**				
Mean ± SD	0.47 ± 0.17	1.11 ± 0.31	0.65 ± 0.32	F=34.37,
				p<0.001
**GBCA exchange rate (10^-2^min^-1^)**				
Mean ± SD	1.39 ± 3.41	1.61 ± 1.39	1.98 ± 3.02	F=0.28,
				p=0.76

a Water exchange rate values in WMH based on data from n=17 (n=6 low WMH burden; n=1 WMH outwith the DW-ASL imaging plane).

### Inter-associations between water exchange rate and DCE-MRI BBB metrics

**Fig. 1:**
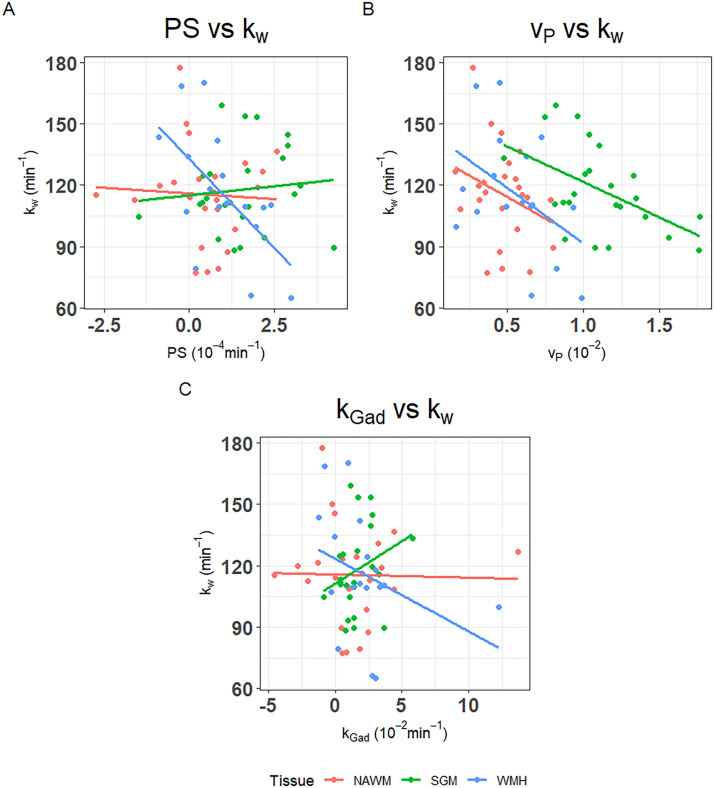
Scatter plots of A) permeability surface area product (*PS*) against water exchange rate (*k_w_*), B) blood plasma volume (*v_P_*) against *k_w_* and C) Gadolinium-based contrast agent exchange rate (*PS*/*v_P_, k_Gad_*) against *k_w_* by tissue type (normal-appearing white matter (NAWM), subcortical grey matter (SGM) and white matter hyperintensities (WMH).

In univariate analyses (Fig. 1, Table 3), patients with higher SGM *k_w_* had lower SGM *v_P_* (B=-34.65, 95% confidence interval (95%CI)=-60.37,-8.93, p=0.01) and tended to have marginally higher SGM *k_Gad_* (B=4.17, 95%CI=-2.23,10.57, p=0.19), but *PS* did not associate with *k_w_* in SGM. Patients with higher NAWM *k_w_* tended to have lower *v_P_* (B=-42.15, 95%CI=-102.77,18.48, p=0.16), but we found no clear associations with *PS* or *k_Gad_*. Patients with higher WMH *k_w_* had lower WMH *PS* (B=-17.34, 95%CI=-29.78,-4.91, p=0.01), and tended to have lower WMH *v_P_* (B=-54.15, 95%CI=-116.22, 7.93, p=0.08) and WMH *k_Gad_* (B=-3.56, 95%CI=-8.73, 1.60, p=0.16). After correcting for WMH volume and age, all associations were attenuated, though the directions of effect were unchanged.

**Table 3:** Linear regressions with water exchange rate (*k_w_*) as the outcome against DCE-MRI BBB metrics: permeability surface area (*PS*), blood plasma volume (*v_P_*) and Gd-based contrast exchange rate (*k_Gad_*=*PS*/*v_P_*) each in a separate model. Multivariable models were adjusted for WMH volume and age.

	Analysis type					
	Univariable			Multivariable		
	B	95% CI	p	B	95% CI	p
*k_w_* against *PS*						
SGM	1.77	-5.71, 9.26	0.63	-0.23	-7.47, 7.01	0.95
NAWM	-1.11	-10.12, 7.89	0.80	-1.48	-10.29, 7.33	0.73
WMH	-17.34	-29.78, -4.91	0.01	-15.31	-28.98, -1.64	0.03
*k_w_* against *v_P_*						
SGM	-34.65	-60.37, -8.93	0.01	-29.40	-55.46, -3.34	0.03
NAWM	-42.15	-102.77, 18.48	0.16	-30.06	-93.32, 33.20	0.33
WMH	-54.15	-116.22, 7.93	0.08	-43.83	-113.88, 26.22	0.20
*k_w_* against *k_Gad_*						
SGM	4.17	-2.23, 10.57	0.19	2.48	-3.92, 8.87	0.43
NAWM	-0.15	-3.24, 2.93	0.92	-0.54	-3.64, 2.57	0.72
WMH	-3.56	-8.73, 1.60	0.16	-2.59	-8.51, 3.34	0.36

### Age associations with BBB metrics

In older participants, we found *k_w_* tended lower across tissues (e.g. SGM: B=-0.74, 95%CI=-1.63, 0.15, p=0.10; Table 4, Figure S2). We did not generally find meaningful associations between age and *PS, v_P_* or *k_Gad_*, though older participants tended to have higher WMH *k_Gad_* (B=0.11, 95%CI=-0.022, 0.25, p=0.09).

**Table 4:** Linear regressions with each BBB metric water exchange rate (*k_w_*), permeability surface area (*PS*), blood plasma volume (*v_P_*) and Gd-based contrast exchange rate (*k_Gad_*) in subcortical grey matter (SGM), normal-appearing white matter (NAWM) and white matter hyperintensities (WMH) assessed in separate models with baseline WMH volume and age as predictors.

	Covariate					
	Percentage WMH volume (%)			Age		
	B	95% CI	p	B	95% CI	p
*k_W_*						
SGM	14.59	1.00, 28.18	0.04	-0.74	-1.63, 0.15	0.10
NAWM	14.45	-1.73, 30.63	0.08	-0.38	-1.44, 0.68	0.47
WMH	19.48	-5.34, 44.30	0.11	-0.97	-2.55, 0.60	0.21
*PS*						
*SGM*	0.40	-0.48, 1.27	0.35	-0.029	-0.086, 0.029	0.31
*NAWM*	0.082	-0.77, 0.94	0.84	-0.0087	-0.065, 0.047	0.75
*WMH*	-0.39	-1.45, 0.66	0.44	0.033	-0.036, 0.10	0.33
*v_P_*						
SGM	-0.16	-0.38,0.055	0.14	0.0016	-0.013, 0.016	0.82
NAWM	-0.074	-0.19, 0.042	0.20	0.00020	-0.0074, 0.0078	0.96
WMH	-0.22	-0.43, -0.011	0.04	-0.0016	-0.015, 0.012	0.81
*k_Gad_*						
SGM	0.68	-0.29, 1.66	0.16	-0.014	-0.078, 0.050	0.66
NAWM	0.79	-1.63, 3.22	0.50	0.050	-0.11, 0.21	0.52
WMH	-0.27	-2.34, 1.80	0.79	0.11	-0.022, 0.25	0.09

### Baseline WMH volume associations with BBB metrics

In participants with higher WMH volumes, across tissues *k_w_* tended higher (e.g. SGM: B=14.59, 95%CI=-1.00,28.18, p=0.04) and *v_P_* lower (e.g. SGM: B=-0.16, 95%CI=-0.38, 0.055, p=0.14). We found no definite associations between WMH volume and *PS* or *k_Gad_*, though in patients with higher WMH volumes SGM and NAWM *PS* and *k_Gad_* tended to be higher while WMH *PS* and *k_Gad_* tended to be lower (Table 4, Figure S3).

### 1-year WMH volume increase associations with BBB metrics

**Fig. 2:**
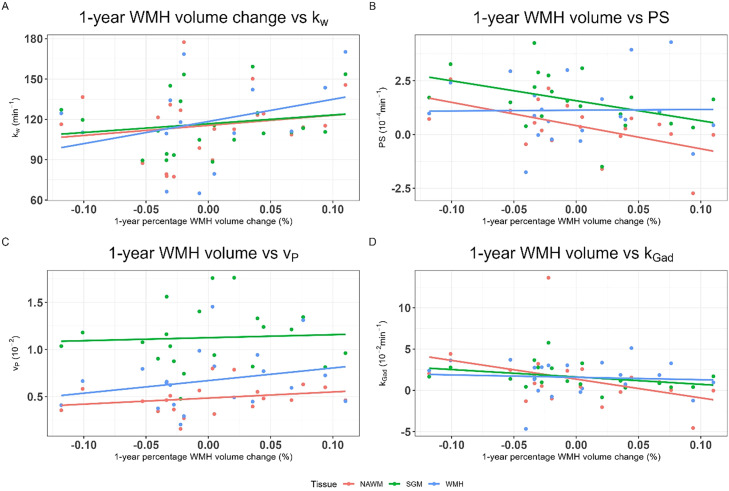
Scatter plots of 1-year WMH volume change against: A) water exchange rate (*k_w_*), B) permeability surface area product (*PS*), C) blood plasma volume (*v_P_*) and D) Gadolinium-based contrast agent exchange rate (*k_Gad_*) by tissue type (normal-appearing white matter (NAWM), subcortical grey matter (SGM) and white matter hyperintensities (WMH).

Patients with greater WMH volume increase at 1-year tended to have higher baseline *k_w_* in all tissues, but were marginally stronger associations for *k_w_* in SGM (B=0.0013, 95%CI=-0.000068, 0.0026, p=0.06) and WMH (B=0.0012, 95%CI=0.00010, 0.0024, p=0.04) than NAWM (B=0.00081, 95%CI=-0.00032, 0.0019, p=0.15) (Fig. 2, Table 5).

**Table 5:** Linear regressions with 1-year WMH volume as the outcome against the BBB metrics water exchange rate (*k_w_*), permeability surface area (*PS*), blood plasma volume (*v_P_*) and Gd-based contrast exchange rate (*k_Gad_*=*PS*/*v_P_*) measured in subcortical grey matter (SGM), normal-appearing white matter (NAWM) and white matter hyperintensities (WMH) in separate models adjusted for baseline WMH volume and age.

	B	95% CI	p
1-year WMH volume against *k_w_*			
SGM	0.0013	-0.000068, 0.0026	0.06
NAWM	0.00081	-0.00032, 0.0019	0.15
WMH	0.0012	0.00010, 0.0024	0.04
1-year WMH volume against *PS*			
SGM	-0.017	-0.038, 0.0052	0.13
NAWM	-0.026	-0.047, -0.0049	0.02
WMH	-0.0019	-0.021, 0.017	0.84
1-year WMH volume against *v_P_*			
SGM	-0.011	-0.10, 0.081	0.81
NAWM	0.056	-0.13, 0.24	0.53
WMH	0.031	-0.072, 0.13	0.54
1-year WMH volume against *k_Gad_*			
SGM	-0.011	-0.031, 0.0083	0.24
NAWM	-0.0061	-0.014, 0.0018	0.12
WMH	-0.0022	-0.015, 0.011	0.72

Patients with greater WMH volume increase at 1-year had lower NAWM *PS* (B=-0.026, 95%CI=-0.047, -0.049, p=0.02) and SGM *PS* tended lower (B=-0.017, 95%CI=-0.038, 0.052, p=0.13), though there was no clear association in WMH *PS* (B=-0.0019, 95%CI=-0.021, 0.017, p=0.84). However, we found no consistent associations between 1-year WMH increase and *v_P_* in any tissue (e.g. SGM: B=-0.011, 95%CI=-0.10, 0.081, p=0.81). SGM and NAWM *k_Gad_* tended lower in patients with greater 1-year WMH volume increase e.g. (SGM: B=-0.011, 95%CI=-0.031, 0.0083, p=0.24), but WMH *k_Gad_* differed little (B=-0.0022, 95%CI=-0.015, 0.011, p=0.72).

## Discussion

We investigated how *k_w_* related to GBCA-derived BBB metrics and differed with WMH severity and change over 1 year in patients with stroke-related SVD. We found participants with higher *k_w_* tended to have higher *k_Gad_* in SGM and lower *v_P_* in SGM and NAWM, suggesting only limited inter-associations between *k_w_* and GBCA-based BBB metrics. While *k_w_* tended to be higher in patients with higher baseline and 1-year increase in WMH volume, we did not find consistent associations between baseline/1-year increase in WMH volume and GBCA-derived BBB metrics.

### Limited inter-associations between water and GBCA BBB function measures

Consistent with previous validation studies, we found only limited associations between water and GBCA exchange metrics. Relatively few studies have assessed how GBCA BBB measures associate with *k_w_* in SVD. In community-dwelling elderly (n=16), Shao et al reported *k_w_* and *K^Trans^* (volume transfer constant from plasma to extracellular space) positively correlated in white matter, the caudate and middle cerebral artery perforator territory, but not the whole brain or grey matter [28]. Meanwhile, *k_w_* and *k_Gad_* only correlated in the MCA perforator territory, medial-temporal lobe and hippocampus. One recent study reported no significant correlations between whole-brain, deep grey matter, NAWM and WMH *k_w_* and *K^trans^* in sporadic SVD (n=30) [14]. By contrast, in CADASIL (n=40) and HTRA1‐related SVD (n=10) respectively higher deep grey matter and whole-brain *k_w_* correlated with lower *K^trans^*. Mirroring these findings in sporadic SVD, we did not generally find consistent associations between *k_w_* and *PS*, except in WMH, where the sample size was reduced by the limited extent and spatial distribution of WMH volume in some patients. Generally, we found SGM, NAWM, and WMH *k_w_* tended higher with lower *PS* after correcting for age and WMH volume, reflecting findings in CADASIL and HTRA1-related SVD [14]. Water and GBCA exchange probe different BBB transport mechanisms [28]. While GBCA-derived metrics assess extravasation of GBCA into the brain parenchyma, *k_w_* is thought to evaluate passive diffusion and active transport of water [16,28], which may provide complementary information on BBB integrity. Finally, lower *v_P_* in sporadic SVD patients with higher *k_w_* is generally consistent with vascular changes [29,30].

### Age and BBB function

While older participants generally had lower *k_w_*, we found only limited evidence of differences in *PS, v_P_* or *k_Gad_*. In line with our findings, several previous studies have reported *k_w_* generally trends lower with older age [31,32]. Notably, in a large cohort study (n=186, age range: 8-92), Shao et al reported grey matter *k_w_* sharply declined with age after 62 [31]. While *PS/k_Gad_* and *v_P_* are typically thought to become respectively higher and lower with age, in SVD conventional associations with BBB metrics may be confounded by existing comorbidities, particularly vascular risk and poor lifestyle factors [30]. As *PS* is defined as the product of permeability and vessel surface area, age- or SVD-related vessel size and density changes may disrupt associations [[33], [34], [35]], with *v_P_* explaining much of the variance in *PS* [36]. Normalising by *v_P_* (*k_Gad_*) accounts for differences in blood volume, but not vessel size, but generally we did not observe substantial differences between models including *k_Gad_* or *PS*. Therefore, methods to assess vessel density should be developed and evaluated to help assess BBB leakage more accurately [[10], [30],^37^].

### Baseline SVD burden and altered BBB function

Patients with sporadic SVD with higher WMH volumes had higher *k_w_*. Findings from previous studies have been mixed. Shao et al found a positive correlation between higher *k_w_* and Fazekas score in community-dwelling elderly at risk of SVD (n=19) [9]. By contrast, one study in sporadic SVD (lacunar stroke/vascular cognitive impairment presumed to relate to SVD, n=30) reported no associations between *k_w_* and WMH volume, nor other SVD markers, including number of lacunes, microbleeds and PVS score [14]. However, the inclusion criteria for sporadic SVD differed from our work (e.g. we scanned patients 1-3 months post-stroke to avoid acute stroke-related BBB effects which could disrupt associations with *k_w_* and did not include patients diagnosed with vascular cognitive impairment). More broadly, higher WMH volume has previously been associated with lower *k_w_* in CADASIL [11,14,38], HTRA1-related SVD [14] and mild cognitive impairment [12]. Previously, CADASIL and sporadic SVD were found to show similar vascular dysfunctions, including GBCA BBB measures, with apparent differences largely reflecting disease severity [39]. Taken together with previous findings of varying associations between *k_w_* and WMH volume in CADASIL vs sporadic SVD [14], this may tentatively suggest associations may vary across the disease course with SVD severity and types [16,40]. However, confirmatory findings in larger cohort studies are required.

We did not find consistent associations between WMH volume and *PS*/*k_Gad_*. However, consistent with previous results [30,39,41], we found *v_P_* generally tended to be lower in patients with higher WMH volume reflecting SVD-related vascular changes. Several studies have found higher BBB leakage associates with higher WMH severity [5,41], but others findings were more mixed [3,30,39,42]. For example, Li et al found higher GM, WM and WMH *K^Trans^* with increasing WMH burden [41]. However, Zhang et al reported lower WMH *K_i_* in patients with higher WMH volume, while NAWM and cortical grey matter *K_i_* (permeability) did not associate with WMH volume [42]. Accurate assessment of subtle BBB leakage, as in SVD, remains challenging due to the limited signal-to-noise ratio, even following gold-standard consensus protocols [3,21], and the previously discussed inability to separate the effects of SVD- and age-related vascular changes [30]. Larger sample sizes (including multicentre collaborations) [39], further harmonisation and methodological development [3,21] are needed to untangle these interacting effects.

### 1-year WMH increase and BBB function associations

In line with our hypothesis, we found higher 1-year WMH volume increase was generally associated with higher *k_w_*, while *PS* and *k_Gad_* tended to be lower. Associations between water exchange and SVD progression have not been widely explored previously [16]. In patients with white matter lesions/chronic ischaemia (n=41), Fujima et al found *k_w_* at 2-year follow-up did not differ in ROIs with versus without WMH progression, though the square difference (relative to mean *k_w_* in age-matched healthy controls with no WMH, n=5) was higher in regions where WMH had progressed [40]. Several pathological processes are implicated in WMH progression, including neuroinflammation, demyelination, axonal loss and gliosis [1]. However, evidence on how AQP4 and vascular dysfunctions, including BBB impairment, interact with disease progression in humans remains scarce [43], therefore robust translational validation studies in clinically relevant models are required to assess how these different effects interact with SVD change [37].

### Strengths/limitations

This work has several strengths. We conducted this work in a subset of patients from an established and extremely well-characterised cohort with extensive contextual data, directly comparing water and GBCA measures of BBB leakage. We used established acquisition and processing protocols for the structural, DW-ASL and DCE-MRI data which were optimised for SVD research. Where available we followed relevant consensus guidelines [3,18]. We included a mix of cortical and lacunar patients to include a broader range of SVD severity while controlling for guidelines stroke prevention, received by all patients, and similar VRFs [17]. To our knowledge, our study is amongst the first studies to investigate how *k_w_* varies with longitudinal SVD change at 1-year. Limitations include the relatively small sample size, as this was an exploratory pilot analysis. We only included patients with sporadic SVD who had lacunar or mild cortical strokes. Not all participants completed scans at 1-year, which may have introduced bias, however retention was very high with over 90% (22/24) completing follow-up. We did not acquire DW-ASL scans at 1-year, therefore we are unable to assess whether change in *k_w_* and SVD severity co-vary. Future studies with larger sample sizes should explore how *k_w_* varies with cognitive, physical and SVD burden changes over longer follow-up durations. Additionally, other markers of SVD e.g. perivascular space volume should be evaluated. The effects of local differences in *k_w_* e.g. adjacent to WMH should also be investigated. Importantly, both DCE-MRI and DW-ASL remain technically challenging, complex imaging techniques, further methodological improvements and validation is needed to improve harmonization [3,44]. Inter-associations with other variables, including local differences in perfusion and diffusivity, should also be evaluated.

## Conclusion

In this small study, we found evidence of associations between DW-ASL and GBCA BBB function metrics. *k_w_* was sensitive to SVD severity and 1-year change, consistent with previous studies using GBCA [5,6,30,45,46], suggesting DW-ASL is a promising imaging marker of subtle BBB dysfunction. However, limited inter-relations between *k_w_* and GBCA-metrics suggest *k_w_* probes different facets of BBB dysfunction than conventional GBCA-metrics. Further studies are required to validate these findings in a larger cohort, to evaluate longitudinal changes in *k_w_* and associations with other disease burden metrics and understand the role of *k_w_* in assessing BBB leakage.

## Funding sources

Funding is gratefully acknowledged from Fondation Leducq Network (16 CVD 05); the UK Dementia Research Institute which receives its funding from DRI Ltd, primarily funded by the UK MRC; Scottish Funding Council through the Scottish Imaging Network, A Platform for Scientific Excellence (SINAPSE) Postdoctoral and Early Career Researcher Exchanges scheme and Stroke Association Post-Doctoral Fellowship (MSS) (SAPDF 23/100007); Row Fogo Charitable Trust Centre for Research into Aging and the Brain; NHS Lothian Research and Development Office (MJT); Mexican National Council of Science and Technology and Anne Rowling Regenerative Neurology Clinic (CAR); Chief Scientist Office of Scotland Clinical Academic Fellowship (UC) (CAF/18/08); Stroke Association Princess Margaret Research Development Fellowship (UC) (2018); Wellcome Trust Translational Neuroscience Ph.D. programme (224912/ Z/21/Z) (DJG); Stroke Association-Garfield Weston Foundation (The Stroke Association Lectureship 2015/04), NHS Research Scotland, and Agnes Parry Endowment at the University of Edinburgh (FND) and US National Institutes of Health (NIH) grants R01NS134712 and R01NS114382 (DJJW). The 3T scanner is funded by the Wellcome Trust (104916/Z/14/Z), Dunhill Trust (R380R/1114), Edinburgh and Lothians Health Foundation (2012/17), Muir Maxwell Research Fund and University of Edinburgh.

## CRediT authorship contribution statement

**Michael S Stringer:** Writing – review & editing, Writing – original draft, Visualization, Project administration, Methodology, Investigation, Funding acquisition, Formal analysis, Conceptualization. **Xingfeng Shao:** Writing – review & editing, Software, Resources, Methodology. **Hedok Lee:** Writing – review & editing, Methodology, Funding acquisition, Conceptualization. **Antoine Vallatos:** Writing – review & editing, Methodology, Funding acquisition, Conceptualization. **Carmen Arteaga-Reyes:** Writing – review & editing, Methodology, Investigation, Data curation. **Una Clancy:** Writing – review & editing, Methodology, Investigation. **Francesca Chappell:** Writing – review & editing, Methodology, Data curation. **Cameron Manning:** Writing – review & editing, Methodology, Formal analysis. **Maria Valdes-Hernandez:** Writing – review & editing, Software, Methodology, Investigation. **Daniela Jaime Garcia:** Writing – review & editing, Methodology, Investigation, Data curation. **Rosalind Brown:** Writing – review & editing, Project administration, Investigation, Data curation. **Fergus N Doubal:** Writing – review & editing, Resources, Methodology, Investigation. **Helene Benveniste:** Writing – review & editing, Supervision, Funding acquisition, Conceptualization. **Michael J Thrippleton:** Writing – review & editing, Supervision, Software, Methodology, Investigation, Funding acquisition, Formal analysis, Conceptualization. **Danny JJ Wang:** Writing – review & editing, Supervision, Software, Resources, Methodology, Investigation, Funding acquisition, Conceptualization. **Joanna M Wardlaw:** Writing – review & editing, Supervision, Resources, Methodology, Investigation, Funding acquisition, Conceptualization.

## Declaration of competing interest

The authors declare the following financial interests/personal relationships which may be considered as potential competing interests:

Michael S Stringer reports financial support was provided by Stroke Association. Carmen Arteaga-Reyes reports financial support was provided by UK Dementia Research Institute. Carmen Arteaga-Reyes reports financial support was provided by Mexican National Council of Humanities Science and Technology. Carmen Arteaga-Reyes reports financial support was provided by Row Fogo Charitable Trust Centre for Research into Ageing and the Brain. Carmen Arteaga-Reyes reports financial support was provided by Anne Rowling Regenerative Neurology Clinic. Una Clancy reports financial support was provided by the Chief Scientist Office of Scotland Clinical Academic Fellowship. Una Clancy reports financial support was provided by the Stroke Association Princess Margaret Research Development Fellowship. Maria Del Carmen Valdes-Hernandez reports financial support was provided by Mrs Gladys Row Fogo Charitable Trust. Daniela Jaime Garcia reports financial support was provided by The Wellcome Trust. Fergus Doubal reports financial support was provided by the Stroke Association-Garfield Weston Foundation. Fergus Doubal reports financial support was provided by NHS Research Scotland. Fergus Doubal reports financial support was provided by the Agnes Parry Endowment at the University of Edinburgh. Danny Wang reports financial support was provided by the US National Institutes of Health. Danny Wang is a co-founder and shareholder of Hura Imaging, Inc. Joanna M Wardlaw reports financial support was provided by Leducq Foundation. Joanna M Wardlaw reports financial support was provided by UK Dementia Research Institute. Joanna M Wardlaw reports financial support was provided by The Stroke Association. Joanna M Wardlaw reports financial support was provided by The Row Fogo Charitable Trust. Joanna M Wardlaw reports financial support was provided by Wellcome Trust. JM Wardlaw is on the Editorial Board of CCCB - Given her role as editorial board member, JM Wardlaw had no involvement in the peer review of this article and had no access to information regarding its peer review. Full responsibility for the editorial process for this article was delegated to another journal editor. If there are other authors, they declare that they have no known competing financial interests or personal relationships that could have appeared to influence the work reported in this paper.

## Appendix Supplementary materials

Image, application 1
