# Supplementary material for: Gadolinium- and water-based blood-brain barrier dysfunction measures in patients with sporadic small vessel disease
Source: Cereb Circ Cogn Behav. 2026 Jan 13;10:100528. doi: 10.1016/j.cccb.2026.100528 (PMC12860635; doi:10.1016/j.cccb.2026.100528)
Supplement: Supplementary file 1 [file mmc1.docx]

**Supplementary Material**

**Figure S1**: Bar charts of blood-brain barrier parameters by tissue type showing: A) water exchange rate (*k_w_*), B) permeability surface area product (*PS*), C) blood plasma volume (*v_P_*), and D) Gadolinium-based contrast exchange rate (*k_Gad_*, calculated as *PS*/*v_P_*). (NAWM=normal-appearing white matter, SGM=subcortical grey matter, WMH=white matter hyperintensities).


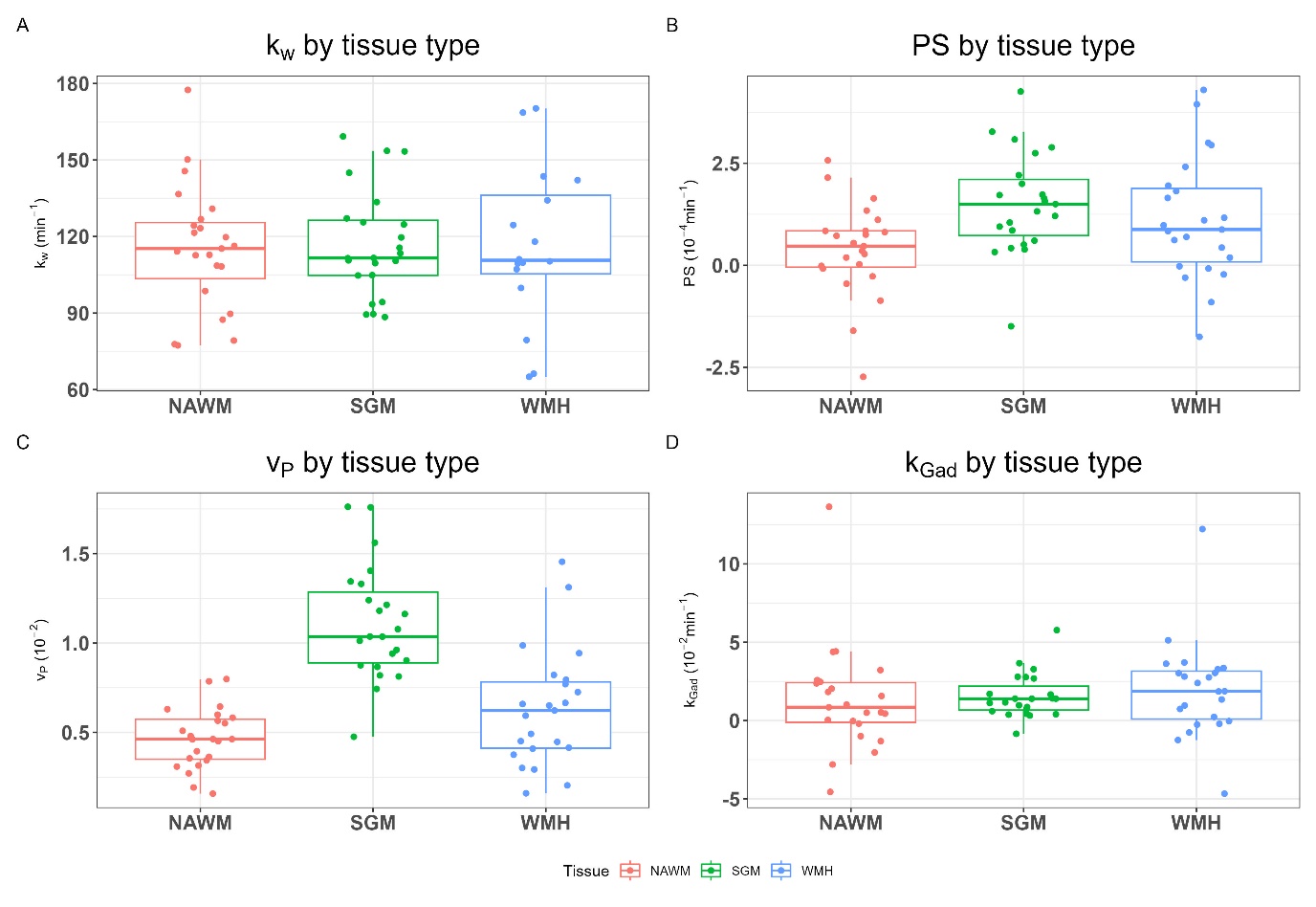


**Figure S2:** Scatter plots of age against: A) water exchange rate (*k_w_*), B) permeability surface area product (*PS*), C) blood plasma volume (*v_P_*) and D) Gadolinium-based contrast agent exchange rate (*k_Gad_*) by tissue type (normal-appearing white matter (NAWM), subcortical grey matter (SGM) and white matter hyperintensities (WMH).**
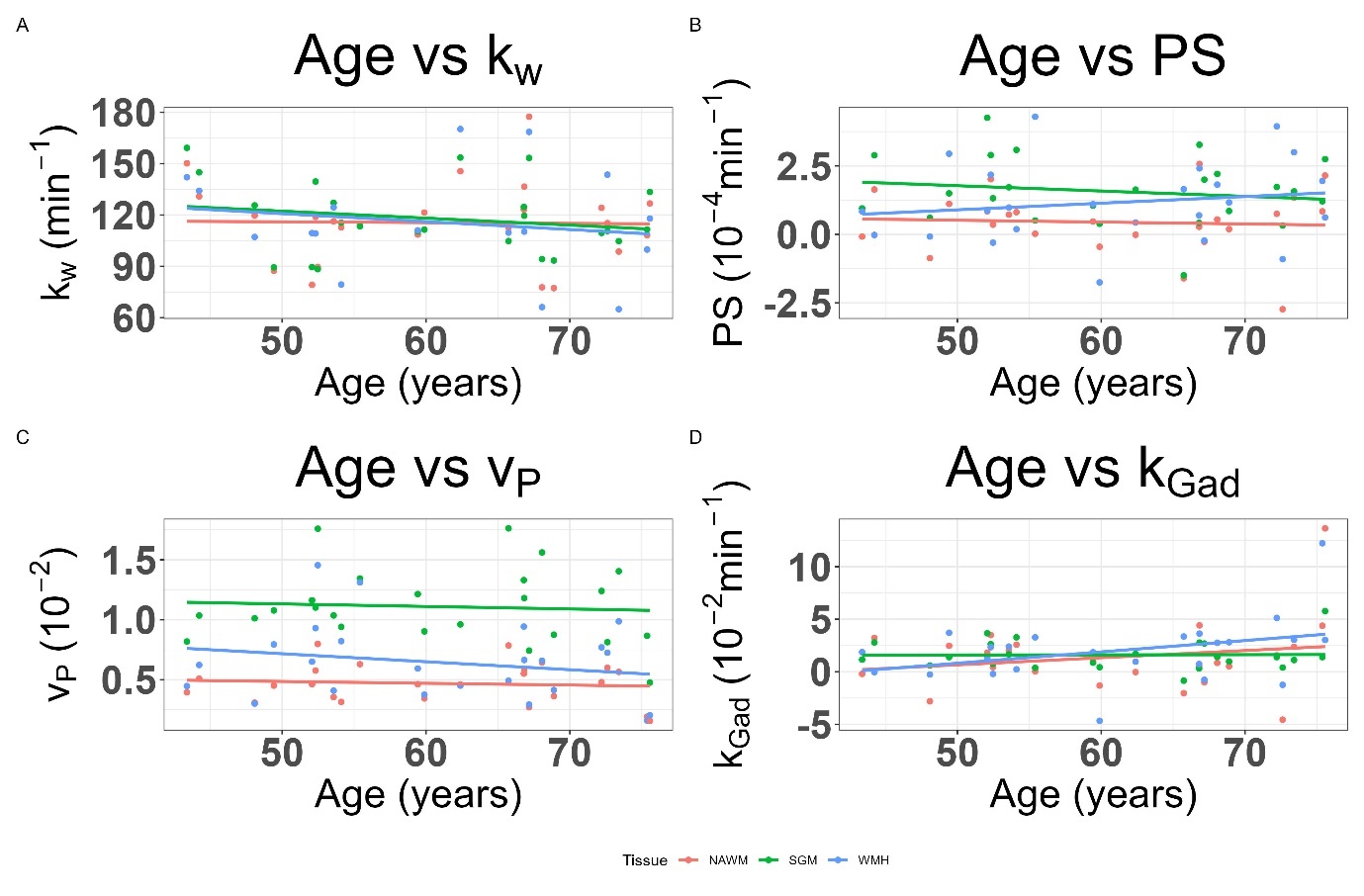
**

**Figure S3:** Scatter plots of percentage baseline WMH volume normalized to ICV (pctWMH volume) against: A) water exchange rate (*k_w_*), B) permeability surface area product (*PS*), C) blood plasma volume (*v_P_*) and D) Gadolinium-based contrast agent exchange rate (*k_Gad_*) by tissue type (normal-appearing white matter (NAWM), subcortical grey matter (SGM) and white matter hyperintensities (WMH).

**
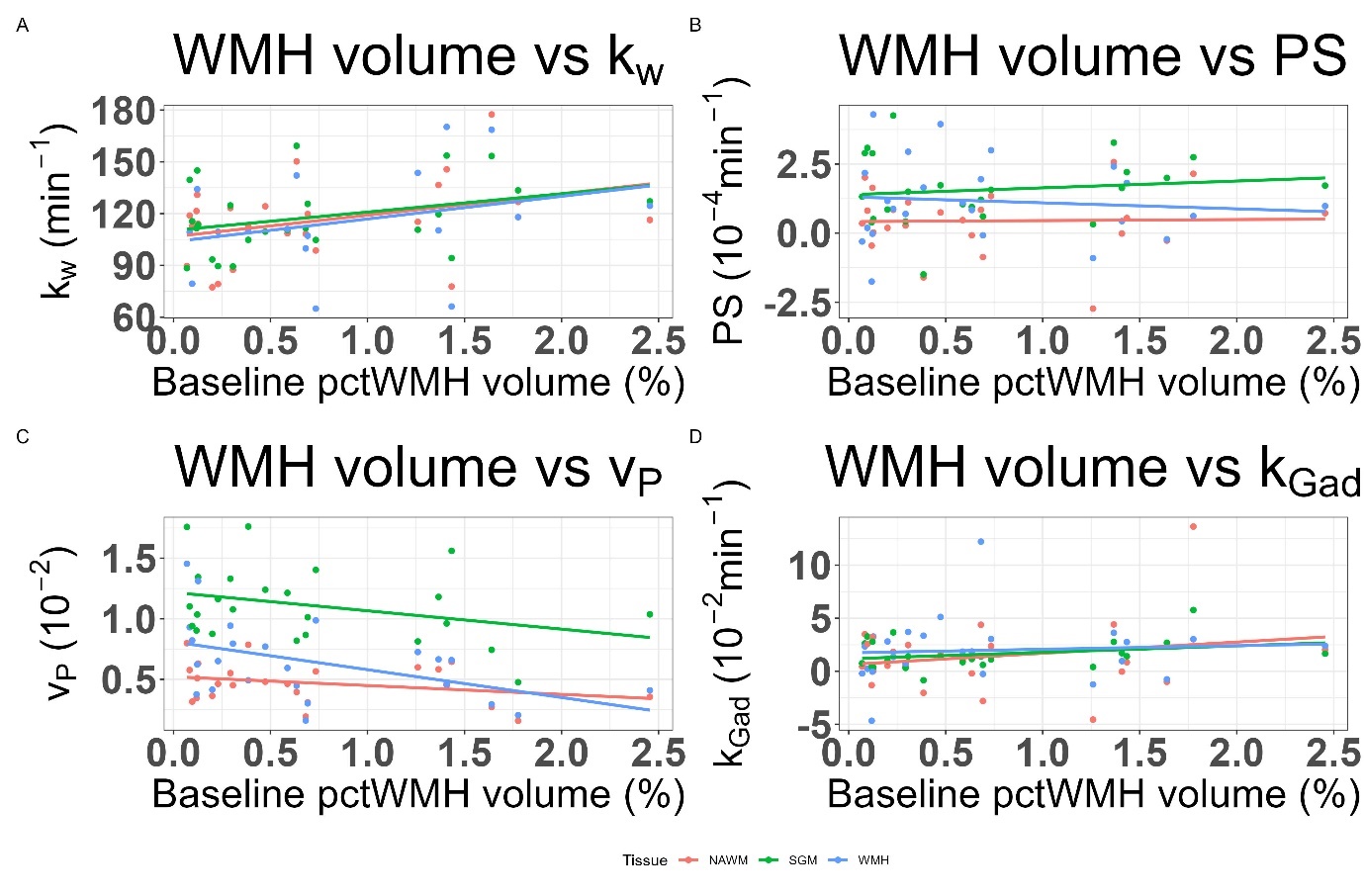
**
